# Disparities in mental health symptoms recovery across race/ethnicity and education level following mild traumatic brain injury

**DOI:** 10.1016/j.dialog.2022.100048

**Published:** 2022-09-24

**Authors:** Rosemay A. Remigio-Baker, Lars D. Hungerford, Su Yeon Lee-Tauler, Jason M. Bailie, Melissa Caswell, Ida Babakhanyan, Mark L. Ettenhofer

**Affiliations:** a Traumatic Brain Injury Center of Excellence (TBICoE), Silver Spring, MD, USA; b General Dynamics Information Technology, Falls Church, VA, USA; c Naval Medical Center San Diego, San Diego, CA, USA; d Naval Hospital Camp Pendleton, Camp Pendleton, CA, USA; e Henry M. Jackson Foundation, Bethesda, MD, USA; f Uniformed Services University of the Health Sciences, Bethesda, MD, USA; g University of California, San Diego, La Jolla, CA, USA

**Keywords:** Race/ethnicity, Depressive Symptoms, Post-traumatic Stress Disorder, Neurobehavioral Signs and Symptoms, Educational Status, Military Health

## Abstract

**Purpose:**

The purpose of this study was to investigate the relationship between race/ethnicity and post-concussive mental health (i.e., depressive, post-traumatic stress disorder [PTSD]) and neurobehavioral symptoms among service members, and whether this association differed by education level.

**Methods:**

The study sample consisted of 524 patients from a multidisciplinary US military outpatient treatment facility for post-concussive symptoms. Poisson regression with robust error variance was utilized to investigate outcome (i.e., clinically-elevated depressive [Patient Health Questionnaire-8 ≥15], PTSD [PTSD Checklist, DSM 5 ≥38] and neurobehavioral [Neurobehavioral Symptom Inventory >75th percentile] symptoms at admission and last follow-up in this cohort study. Modification by education level (low [no college degree] vs. high [associate's degree or higher]) was additionally evaluated.

**Results:**

The relationship between race/ethnicity and mental health/neurobehavioral symptoms varied by education level (p-interaction: depressive symptoms = 0.002, PTSD symptoms = 0.035, neurobehavioral symptoms = 0.040). Specifically, non-Whites were at a significantly higher prevalence for clinically-elevated depressive symptoms post-treatment than Whites, but only among those with higher education level (PR = 2.22, CI = 1.37–3.59). A similar trend was demonstrated for PTSD and neurobehavioral symptoms.

**Conclusion:**

Military healthcare may need to increase depression-focused treatment options that are acceptable for racial/ethnic minority patients, particularly those with higher education, while they are recovering from comorbid traumatic brain injury.

## Highlights

• Race differences in mental health recovery post-TBI are found among service members
• The relationship between race and mental health symptoms varied by education level
• Highly-educated minorities had higher levels of depressive symptoms posttreatment
• Neurobehavioral symptoms posttreatment increased more in highly-educated minorities
• Among neurobehavioral symptoms, affective symptoms were most affected by race

## 1 Introduction

An estimated 1.5 million Americans sustain a traumatic brain injury (TBI) each year, many of whom experience long-term disability including mental health issues such as depression and post-traumatic stress disorder (PTSD) [1,2]. The occupational hazards of the military (e.g., combat training, deployments) play a significant role in the elevated risk of service members (SMs) for sustaining a TBI, with more than 450,000 SM diagnosed since 2000, 82.2% of which injuries are mild in severity (i.e., concussion) [3]. Deployments, such as those during Operations Enduring Freedom and Iraqi Freedom, have not only contributed to an increase in the prevalence of concussions, but also to the psychological toll of these engagements on SMs. Since September 11, 2001, the prevalence of depression and PTSD has escalated to approximately 14% to 16% of the military population [4,5].

As US SMs are predominantly of non-Hispanic White background, it is not surprising that most studies on concussion within the military evaluate samples of predominantly non-Hispanic White participants. As such, although findings from these studies provide a better understanding of post-concussive symptom recovery among SMs, they may not generalize to racial/ethnic minorities. Between 1980 and 2017, the percentage of racial/ethnic minorities in active duty forces rose from 36 to 43% [6]. It is, thus, critical to better understand and characterize the potential risk for poor neuropsychological outcome of racial/ethnic minorities in the military to develop more personalized care to optimize outcomes.

Race/ethnic disparities in mental health are well documented in literature. Racial/ethnic minorities who sustain a TBI, particularly Hispanics, report more psychiatric symptoms, physical limitations and cognitive deficits post-TBI compared to non-Hispanic Whites [7,8]. In a nationwide multicenter study, African Americans in general are also more likely to report depression compared to non-Hispanic Whites [9]. Additionally, African Americans report more severe PTSD symptoms than other racial groups 12 months after sustaining a TBI [10]. Unfortunately, despite having more severe psychiatric symptoms, minorities overall are less likely to seek treatment for mental health [11,12] or report a psychiatric diagnosis [13]. Failure to receive appropriate treatment may exacerbate symptoms [14,15] and explain why racial/ethnic minorities who did receive a psychiatric diagnosis have more persistent symptomatology [16] that are more severe and debilitating than that of non-Hispanic Whites [17]. Consistent with more severe and debilitating psychiatric disorders, African Americans are shown to have worse psychosocial outcomes than non-Hispanic Whites 1 year after a concussion [18]. Many studies on veterans demonstrate significantly higher rates of PTSD among racial/ethnic minorities compared to non-Latinx White. The elevated prevalence rates for PTSD among Black and Hispanic (versus White) Vietnam veterans is found to be explained by greater exposure to war-zone stress [19]. An investigation of records from Iraq and Afghanistan veterans enrolled in VA care finds Asian/Pacific Islander women and Black men to more likely screen positive for PTSD compared to their White counterparts [20]. In addition to such disparities, racial/ethnic minority (versus White) veterans with PTSD are more likely to have a poor course of recovery from PTSD. [21].

Current literature also shows racial/ethnic disparities in TBI-related treatment access [[22], [23], [24], [25], [26], [27]]. In a systematic review of race/ethnic differences in post-TBI outcomes, African Americans and Hispanics are found more likely to report poor functional outcomes post-injury [22]. Among studies evaluating the facilities to which patients are discharged after initial emergency department evaluation for TBI, non-Whites (primarily Blacks and Hispanics) are less likely to be discharged to inpatient rehabilitation than Whites, even after accounting for severity of head injury [[23], [24], [25]]. Further, compared to non-Hispanic Whites, African American and Hispanic TBI patients are twice as likely to be discharged home, with African Americans less likely to be discharged to an assisted living facility even after accounting for functional status at discharge, length of stay, insurance coverage and a comorbidity index [25]. These findings are supported by Fuentes et al. who shows that race/ethnic minorities are admitted to acute TBI rehabilitation at a significantly slower rate than non-Hispanic Whites, though injury severity is similar [28]. Other studies show that, as a group, non-Hispanic Black and Hispanic patients receive less follow-up care and rehabilitation following a TBI compared to Non-Hispanic White patients [[25], [26], [27]]. Additionally, one study shows that racial/ethnic minorities receive fewer therapy services (e.g. physical therapy, occupational therapy, speech-language pathology and psychotherapy) in rehabilitative care than non-Hispanic Whites [29]. Such unequal access to care may explain, in part, race/ethnic differences in recovery among concussed patients. Although these studies have predominantly evaluated patients with moderate to severe TBI, the reasons for existing race/ethnic disparities may be similar regardless of severity. Identification of target populations at greater risk for poor prognosis may help attenuate the long-term burden of chronic mental health symptoms through provision of a more culturally-effective treatment approach and early intervention.

Little is known of the impact of education level on the association between race/ethnicity and mental health outcomes. The combination of lower education and other risk factors associated with race/ethnicity may adversely affect depressive and PTSD symptomatology. Studies show that racial/ethnic minorities have lower health literacy than Whites. This is not a reflection of differences in intelligence, but possibly a measure of acculturation whereby race/ethnic minorities, who may be immigrants or have low English proficiency, may be less versed on navigating through the healthcare system to receive adequate treatment. Cultural differences may also reflect disparities in the level of stigma within race/ethnic groups [30]. Individuals from minoritized groups experience more stigma or have more biases against mental illness [31]. This may explain, in part, disparities in severity of mental health disorders and treatment seeking. Thus, a lower level of education combined with race/ethnicity-related factors, such as less acculturation and/or culturally-tied stigma against mental illness among racial/ethnic minorities, may jointly have adverse consequences in addressing mental health needs, thereby magnifying mental health symptoms among those with lower education.

The primary objectives of this study are to investigate whether there are differences in the level of depressive and PTSD symptomatology post-concussion (the mild and most common form of TBI) by race/ethnicity among SMs being treated in a TBI clinic, and whether this relationship varies by the level of highest education attained. As neurobehavioral symptoms include components of affective symptoms (e.g., feeling depressed or sad; feeling anxious or tense) that are related to mental health issues, as secondary objectives, we additionally evaluate the contribution of race/ethnicity on post-concussive neurobehavioral symptoms and whether this varied by education level. We hypothesize that racial/ethnic minority SMs will have greater depressive, PTSD and neurobehavioral symptoms compared to non-Hispanic White SMs and that these associations will be greater among those with lower education level.

## 2 Materials and methods

### 2.1 Participants

The study participants consisted of patients with a history of concussion (median time since injury = 1.7 years [IQR = 0.2–7.7] prior to the initial clinical visit) receiving treatment between January 2017 and January 2020 from a multidisciplinary TBI treatment program at two large military treatment facilities in the Southwestern US. The available rehabilitative treatments from these sites included: occupational, physical, speech, cognitive and music therapy; headache/pain treatment; and behavioral therapy to name a few. Data was obtained from the Naval Medical Center San Diego IRB-approved clinical data registry where, upon enrollment at the facility, patients agreed to have their data used for research. Patients who agreed to participate were provided with the Project Information Sheet and providers were available to answer any questions. A waiver of informed consent applied for this study.

**Fig. 1:**
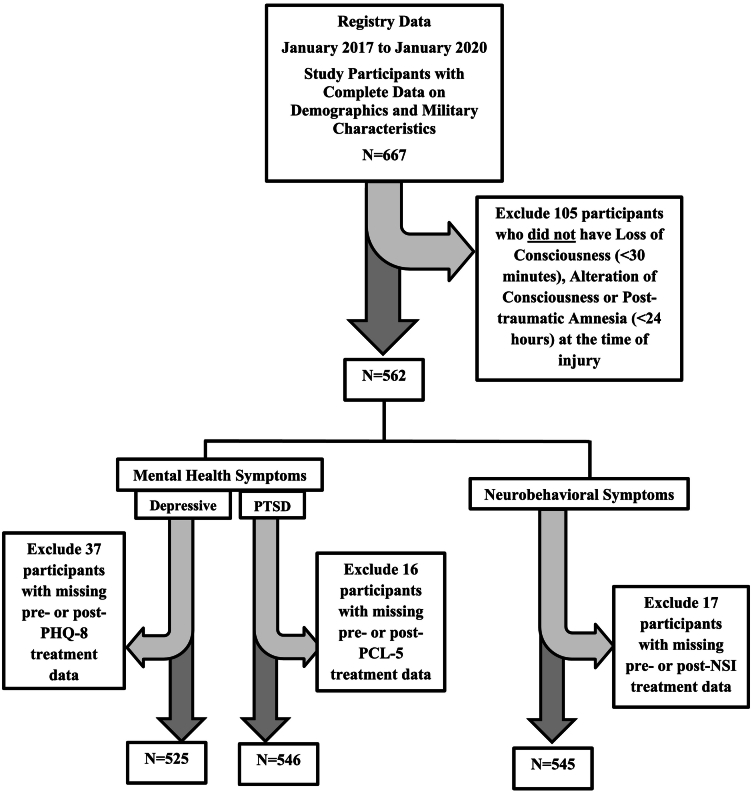
Study Sample Size Flowchart. Abbreviation: PHQ-8 = Patient Health Questionnaire (8 items); PCL-5 = Post-traumatic Stress Disorder Checklist, DSM-V; NSI=Neurobehavioral Symptom Inventory.

Study participants included those with non-penetrating head injury and with loss of consciousness of <30 min, alteration of consciousness at the time of injury and/or post-traumatic amnesia of <24 h (as assessed using a structured interview of the Ohio State University TBI Identification Method [32]) (*n* = 562). There were 37 participants excluded from analyses of depressive symptoms as the outcome (*n* = 525), 16 when assessing PTSD symptoms as the outcome (*n* = 546), and 17 when assessing neurobehavioral symptoms as the outcome (*n* = 545) (Fig. 1). The median days from pre- to post-treatment was 109 days (IQR = 62–179) for all participants, 99 days (IQR = 57–163) for non-Hispanic White participants and 125 days (IQR = 74–224) for other race/ethnic groups.

### 2.2 Measures

#### 2.2.1 Main variables

Self-identified race/ethnicity served as the main independent variable and was dichotomized into non-Hispanic White (or White) and non-White (Black/African American, Hispanic/Latino, Asian, Native Hawaiian/Pacific Islander, and American Indian/Alaska Native). Dichotomization of this variable was used due to the limited sample size of participants defined as “non-White”. As it was understood that *race* and *ethnicity* were two separate key terms (i.e., *race* defined as “the social construction and categorization of people based on perceived shared physical traits that result in the maintenance of a sociopolitical hierarchy, while *ethnicity* was a particular type of culture… related to common ancestry and shared history” [33]), this differentiation was particularly important when interpreting the results using these dichotomized groups along with the use of the term “race/ethnicity” throughout this paper.

There were two primary outcomes, depressive and PTSD symptoms, with neurobehavioral symptoms serving as secondary outcomes. Using the 8-item Patient Health Questionnaire (PHQ-8), depressive symptoms over the past 2 weeks from the time of data collection were assessed. Responses to being bothered by any symptoms ranged from 0 (“Not at all”) to 3 (“Nearly every day”). The PHQ-8 was a well-established instrument to assess depressive symptoms with good internal consistency (Cronbach alpha = 0.82) and good convergent validity [34]. To evaluate PTSD symptoms, the 20-item PTSD Checklist for DSM-5 (PCL-5) was utilized, assessing how participants were bothered by their symptoms in the past month [35]. Responses ranged from 0 (“Not at all”) to 4 (“Extremely”). The PCL-5 had good test-retest reliability (*r* = 0.82), convergent validity (*r* with other PTSD measures = 0.74–0.85), discriminant validity (*r* = 0.31–0.60) when compared with measures of related constructs, and a high level of internal consistency (Cronbach's alpha = 0.94) [36]. The 22-item Neurobehavioral Symptom Inventory (NSI) [37] was used to evaluate neurobehavioral symptoms in the past 2 weeks, in total and by subdomains (e.g., cognitive, somatosensory, vestibular and affective). It had good test-retest reliability (*r* *=* 0.78–0.94), high internal consistency (total alpha = 0.95; subscale alpha = 0.88–0.92) and acceptable convergent validity (*r* = 0.41) [38]. Responses ranged from 0 to 4 and included ‘none,’ ‘mild,’ ‘moderate,’ ‘severe,’ ‘very severe’. Data for PHQ-8, PCL-5 and NSI were collected at intake (defined as pre-treatment) and at the last available visit (defined as post-treatment).

To assess interaction by education level, we utilized the highest education attained which included obtaining a high school equivalency diploma (GED)/high school diploma, some college, or an associate's, bachelor's, master's or doctoral degree. The level of education was dichotomized into lower education (defined as having up to some college) and higher education (defined as having an associate's degree or higher). This chosen cut-off was in consideration of the sample size needed to adequately evaluate modification, while still maintaining a meaningful cut point (i.e., college degree vs. non-college degree-earning education).

#### 2.2.2 Covariates

Additional covariates included demographic (i.e., age, gender, marital status, primary language) and military (i.e., rank, branch of service, deployment history [yes/no], combat deployment history [none/1+], number of years in active duty) characteristics. These were assessed for potential confounding. The severity of pre-treatment symptoms and the length of treatment time were also analyzed.

### 2.3 Statistical analyses

Covariates were evaluated against race/ethnicity using chi-squared tests for categorical variables and *t*-tests for continuous variables assumed to be normally distributed. The association between race/ethnicity and continuous variables assumed to be non-normally distributed was assessed using the Wilcoxon-Mann-Whitney test. Poisson regression with robust error variance was utilized to determine the prevalence of clinically-elevated depressive (PHQ-8 ≥15) [39], PTSD (PCL-5 ≥38) [40] and neurobehavioral (NSI at or above the 75th percentile score [41]) symptoms before and after treatment, as well as clinically-relevant change (pre- to post-treatment) in depressive (increase/decrease in PHQ-8 ≥5), PTSD (increase/decrease PCL-5 ≥8) and neurobehavioral (increase/decrease in NSI ≥7) symptom score [42,43]. For these analyses, the generalized linear model command was utilized with a “modified Poisson” approach and a log-binomial link function to estimate the prevalence ratio (PR). The robust option was included to estimate robust error variances [44]. We additionally evaluated clinically-elevated pre- and post-treatment neurobehavioral symptoms by subdomains (i.e., cognitive, somatosensory, vestibular, and affective) to evaluate whether associations between race/ethnicity and neurobehavioral symptoms may be driven by affective symptoms. Assessment of interaction between race/ethnicity (White vs. non-White) and education level (lower vs. higher) was conducted for all analyses. Models were adjusted for confounders found significantly related to race/ethnicity (Table 1**)**. The main effects and interaction significance were based on a *p*-value of <0.05, and pairwise deletion was used to handle missing data. Analyses were conducted using Stata statistical software, release v.15 (StataCorp, 2017, College Station, TX).

**Table 1:** Sample Characteristics, Overall and by Race/ethnicity (White vs. Non-white).

Characteristics	All (*n* = 546)	Race/ethnicity		
		White (*n* = 361)	Non-white (*n* = 185)	p
Site, n (%)				0.045⁎
Camp Pendleton	295 (54.0)	184 (51.0)	111 (60.0)	
NMCSD	251 (46.0)	177 (49.0)	74 (40.0)	
Age in years, mean (SD)	32.3 (8.7)	32.5 (8.9)	31.8 (8.5)	0.343
Gender, n (%)				0.570
Female	62 (11.4)	39 (10.8)	23 (12.4)	
Male	484 (88.6)	322 (89.2)	162 (87.6)	
Race/ethnicity, n (%)				–
White (Non-Hispanic)	361 (66.1)	361 (100)	–	
Non-White	185 (33.9)	–	185 (100)	
Black/African American	53 (9.7)	–	53 (9.7)	
Hispanic/Latino	84 (15.4)	–	84 (15.4)	
Asian	23 (4.2)	–	23 (4.2)	
Native Hawaiian/Pacific Islander	16 (2.9)	–	16 (2.9)	
American Indian/Alaska Native	9 (1.7)	–	9 (1.7)	
Marital Status, n (%)				0.036⁎
Single	142 (26.0)	87 (24.1)	55 (29.7)	
Married	371 (68.0)	246 (68.1)	125 (67.6)	
Divorced/Separated	33 (6.0)	28 (7.8)	5 (2.7)	
Education Level, n (%)				0.018⁎
Lower	368 (67.4)	231 (64.0)	137 (74.1)	
High School Diploma	181 (33.2)	108 (29.9)	73 (39.5)	
GED	3 (0.6)	1 (0.3)	2 (1.1)	
Some College	184 (33.7)	122 (33.8)	62 (33.5)	
Higher	178 (32.6)	130 (36.0)	48 (26.0)	
Associate's Degree	42 (7.7)	32 (8.9)	10 (5.4)	
Bachelor's Degree	96 (17.6)	67,918.6)	29 (15.7)	
Master's Degree	34 (6.2)	26 (7.2)	8 (4.3)	
Doctoral Degree	6 (1.0)	5 (1.4)	1 (0.5)	
Primary Language, n (%)				<0.001⁎
English	522 (95.6)	360 (99.7)	162 (87.6)	
Non-English^a^	24 (4.4)	1 (0.3)	23 (12.5)	
Branch of Service, n (%)				0.001⁎
Navy	251 (46.0)	184 (51.0)	67 (36.2)	
Marines	272 (49.8)	162 (44.9)	110 (59.5)	
Army	14 (2.6)	6 (1.7)	8 (4.3)	
Air Force	8 (1.5)	8 (2.2)	0 (0)	
Coast Guard	1 (0.2)	1 (0.3)	0 (0)	
Rank, n (%)				0.036⁎
Junior Enlisted	80 (14.7)	47 (13.0)	33 (17.8)	
NCO's	211 (38.6)	136 (37.7)	75 (40.5)	
Staff NCO's	184 (33.7)	121 (33.5)	63 (34.1)	
Officers	71 (13.0)	57 (15.8)	14 (7.6)	
History of Deployment, n (%)				0.077
No	237 (43.4)	174 (40.7)	90 (48.7)	
Yes	309 (56.6)	214 (59.3)	95 (51.4)	
Number of Deployment, median (IQR), Range	0.6 (0.5)	0.6 (0.5)	0.5 (0.5)	0.077
Length of Active Duty in years, mean (SD)	11.6 (8.2)	11.9 (8.2)	11 (8.2)	0.222
Number of Days in the Study, median (IQR)	109 (62, 179)	99 (57, 163)	125 (74, 224)	0.002⁎
Pre-treatment Outcome Measure Scores, mean (SD)				
PHQ-8	11.4 (6.1)	10.8 (5.9)	12.5 (6.4)	0.002⁎
PCL-5	31.0 (19.8)	28.3 (18.6)	36.1 (21.0)	<0.001⁎
NSI	35.5 (16.5)	34.3 (15.9)	37.8 (17.5)	0.019⁎

Abbreviations: NMCSD = Naval Medical Center San Diego; IQR = Interquartile Range; SD=Standard Deviation; PHQ-8 = Patient Health Questionnaire, 8 items; PCL-5 = Post-traumatic Stress Disorder Checklist, DSM-5; NSI=Neurobehavioral Symptom Inventory.

⁎ Significant *p*-value <0.05.

a 1 participant (Non-White) was bilingual at birth.

## 3 Results

Table 1 summarized the characteristics of the study population, overall and by race/ethnicity. Our sample consisted of 34% non-Whites, which was consistent with the proportion in the general military population. When stratified by race/ethnicity, non-Whites were lower ranking, more likely to be single, less likely to have a college degree as defined by our study, and had more days in the treatment program and greater depressive, PTSD and neurobehavioral symptoms pre-treatment compared to Whites. Among non-Whites, those with higher education level were significantly more likely to have clinically-elevated depressive symptoms post-treatment compared to those with lower education. No such relationship was found significant among Whites.

**Fig. 2:**
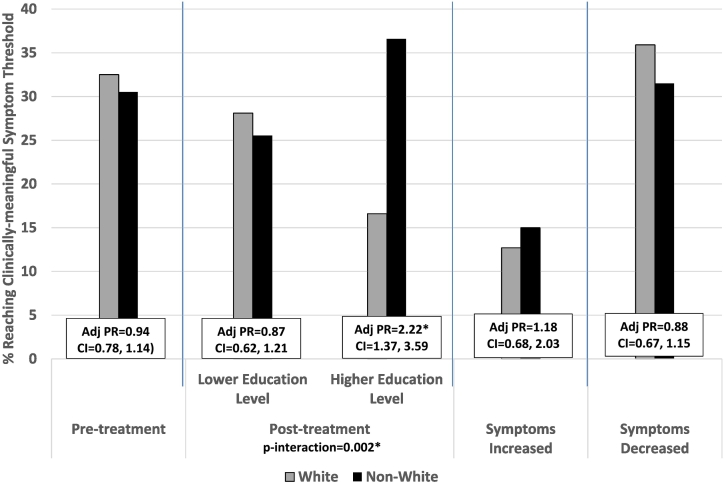
Percent of Clinically-meaningful Depressive Symptom Threshold and Their Adjusted Prevalence Ratio Stratified by Race/ethnicity. Abbreviation: Adj PR = Adjusted Prevalence Ratio; CI = 95% Confidence Interval. *Significant main effect p-value <0.05 Adjusted Prevalence Ratio (Adj PR): Adjusted for marital status, education level, primary language, branch of service, rank, number of days in the study, level of depressive symptoms at intake.

**Fig. 3:**
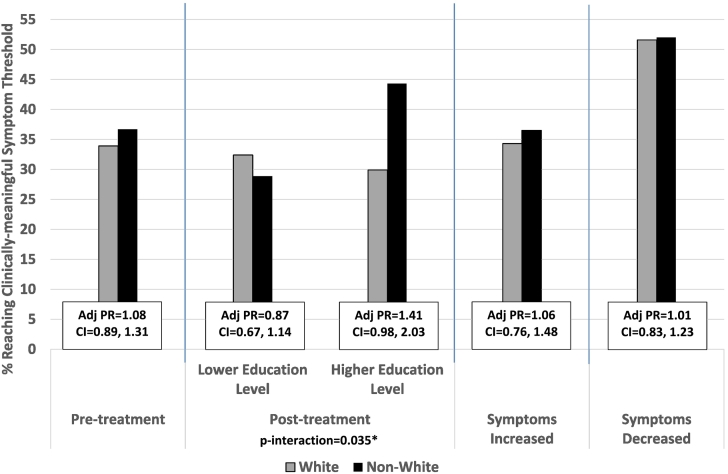
Percent Reaching Clinically-meaningful PTSD Symptom Threshold and Their Adjusted Prevalence Ratio Stratified by Race/ethnicity. Abbreviation: Adj PR = Adjusted Prevalence Ratio; CI = 95% Confidence Interval; PTSD=Post-traumatic Stress Disorder. *Significant interaction p-value <0.05 Adjusted Prevalence Ratio (Adj PR): Adjusted for marital status, education level, primary language, branch of service, rank, number of days in the study, level of PTSD symptoms at intake.

**Supplemental Table 1** illustrated the percentage of participants who reached a clinically-meaningful threshold (i.e., clinically-elevated pre- and post-treatment symptoms and clinically-relevant change in symptoms) for each mental health outcome by race/ethnicity, as well as the PR of the relationship between race/ethnicity and reaching these threshold scores. Interaction *p*-values for each comparison were also included. There were no significant main effects by race/ethnicity in these outcomes; however, the relationship between race/ethnicity and post-treatment clinically-elevated depressive and PTSD symptoms were found to vary by education level (Fig. 2 [p-interaction = 0.002] and Fig. 3 [p-interaction = 0.035], respectively**)**. Significant main effects were only found for depressive symptoms whereby non-Whites (vs. Whites) were more likely to have clinically-elevated post-treatment depressive symptoms, but only among those with higher education (PR = 2.22, CI-1.37-3.59) (See Fig. 2).

**Fig. 4:**
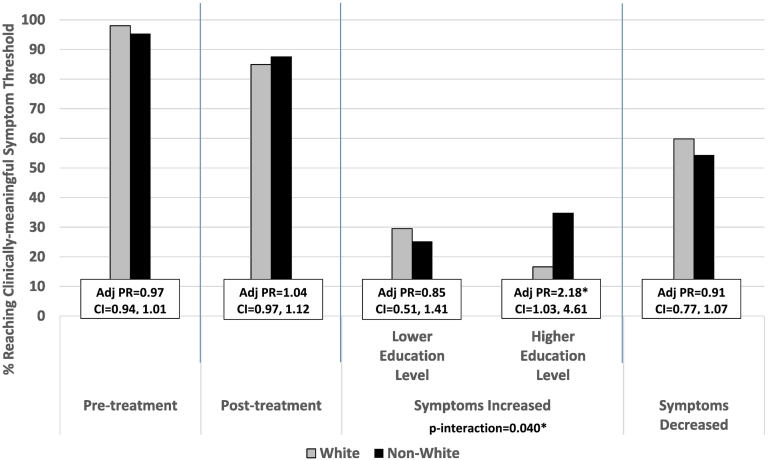
Percent of Clinically-meaningful Neurobehavioral Symptom Threshold and Their Adjusted Prevalence Ratio Stratified by Race/ethnicity. Abbreviation: Adj PR = Adjusted Prevalence Ratio; CI = 95% Confidence Interval. *Significant main effect p-value <0.05 Adjusted Prevalence Ratio (Adj PR): Adjusted for marital status, education level, primary language, branch of service, rank, number of days in the study, level of neurobehavioral symptoms at intake.

**Supplemental Table 2** summarized the results evaluating race/ethnicity against reaching a clinically-meaningful threshold for neurobehavioral symptoms, and whether this relationship varied by education level. There were 30% of non-Whites compared to 24.1% of Whites who had a clinically-relevant increase in neurobehavioral symptoms where significant difference was found only among those with higher education (p-interaction = 0.040, PR = 2.18, CI = 1.03–4.61, Fig. 4). When evaluated by subdomains of neurobehavioral symptoms, non-Whites were significantly less likely to have clinically-elevated affective symptoms at pre-treatment compared to Whites (PR = 0.94, CI = 0.90–0.99). No other significant main effects or interaction by education was found.

## 4 Discussion

Racial/ethnic disparities in the recovery of depressive and neurobehavioral symptoms varied by the level of education among SMs who received rehabilitation treatment for their concussion. Specifically, non-Whites had a higher proportion with clinically-elevated depressive symptoms post-treatment compared to Whites, but only among those with higher education. Although the association between race/ethnicity and clinically-elevated PTSD symptoms post-treatment significantly varied by education, the relationship within each level of education was not found significant. Non-White study participants were also more likely to have a clinically-relevant increase in overall neurobehavioral symptoms compared to their counterparts, but also only among those with higher education level.

Although our hypothesis was supported by the study findings, showing non-White SMs with greater mental health symptoms (i.e., depressive and PTSD) post-treatment compared to White SMs, it was not supported in that we found this association among those with higher rather than lower level of education. However, this specific effect of *higher* education in military personnel was not entirely counter intuitive. While it was beyond the scope of this study to understand factors associated with racial/ethnic disparities in neuropsychological recovery, it was possible that personal and cultural beliefs may be connected to the reluctance to seek professional treatment. As previously mentioned, the differences by race/ethnicity in our study might be explained by self-stigma and/or the extent of negative attitudes towards mental health disorders (such as depression and its treatment) [[45], [46], [47]]. Non-Whites tend to have higher self-stigma regarding depression compared to Whites, and were less likely to seek mental health treatment, or continue with treatment once it had begun [46,48]. This might lead to the worsening of symptoms. Additionally, there was fear of being labeled as “weak” due to mental health disorders [49], which might be heightened in race/ethnic minorities [50]. Such fear might be particularly amplified among those at higher positions or seeking higher positions who might see this perception of weakness as having a negative consequence on their career (e.g., hindering progression). Moreso, race/ethnic disparities in career promotion in the military might further worsen this fear [51]. These might lead to the postponement of necessary follow-ups or completing an insufficient number of follow-ups, whereby the last follow-up might be a reflection of worsened symptoms due to inadequate treatment or follow-up. This might explain the finding of modification by education level at post-treatment, but not pre-treatment. This potential rise in mental health symptoms might not have reached clinically-relevant levels and, thus, no significance was found when assessing clinically-relevant increases in depressive or PTSD symptoms. With regards to seeing a significant association only among those with higher education, individuals at higher position or seeking higher positions might be likely those who also had higher education. This was consistent with our study findings that showed a higher level of education significantly related to a higher ranking for both race/ethnic groups. Taken together, among those with higher education, non-White SMs might be less likely than White SMs to seek treatment to protect their professional standing and opportunities for promotion in reaction to unconscious bias, leading to worsened symptoms.

Institutional discrimination in healthcare might also contribute to the disparities between non-White SMs and White SMs (e.g., clinician bias, dismissal or misattribution of reported symptoms by non-White participants, microaggressions towards non-White participants) [52]. A review of literature found that race/ethnic disparities in healthcare could be linked to both the providers' beliefs and behaviors towards race/ethnic minorities [53]. Further, Perez-Stable and El-Toukhy reported that poor quality patient-clinician communication was related to patients being a racial/ethnic minority and to providers being less culturally competent and holding unconscious biases [54]. In a study by van Ryn and Burke, physicians were reported to view Black patients to be “less kind, congenial, intelligent, and educated,” even after accounting for age, sex socioeconomic status, overall health, and social support [55]. Schulman et al. also provided evidence of differing provider perception based on the race/ethnicity of their patient regardless of their clinical characteristics [56]. The National Academics of Sciences, Engineering and Medicine also reported that racial/ethnic minority veterans were more likely to “experience barriers to accessing VA mental health services, to not feel welcome in VA facilities, and to have worse health care experiences” [57]. Although these issues could not be directly determined in the current study, studies of institutional discrimination were believed to result in health disparities in many settings, including within the US military [52,58]. The type and frequency of treatment (previous and/or current), although not available for the current study, might provide further information as to the race/ethnic disparities that existed with mental health treatment (e.g., whether different race/ethnic groups receive differential treatments). Future studies were warranted to investigate the mechanisms through which non-White SMs with higher education might experience worsening mental health symptoms relative to their White counterparts.

Although poorer mental health was associated with lower socioeconomic status, higher education attainment did not necessarily equate to less stigma against mental health illness and treatment [59]. Such stigma might be deeply rooted in culture and unconscious bias (whether by an individual or a society). Thus, interventions such as strengthening mental health literacy and culturally effective education [60], which might address and attenuate stigma against mental illness, might help improve mental health recovery among highly-educated non-White concussed patients.

Another plausible explanation for our findings was that during treatment patients begin having a better understanding of their symptoms and might be more likely to report them. Thus, for some patients, an increase in symptoms might reflect a better understanding of concussion recovery, particularly with mental health, and increased willingness to divulge these symptoms despite perceived stigmas. Such investigation was not possible in the current study; however, future studies might provide this clarification as well as evaluate the means to capture symptom progression accounting for increasing health literacy. Higher education might allow for faster absorption and retention of new information, which might result in improvement in reporting of symptoms [61], and, thus, reflect as greater symptomatology. The impact of health literacy might transcend beyond improvement in understanding of health symptoms; it might also result in a better understanding of how to navigate the healthcare system. As non-Whites demonstrated to have lower health literacy compared to Whites [62], this might also explain, in part, the race/ethnic difference in post-treatment depressive symptomatology among those with higher education.

The differences in depressive and neurobehavioral symptoms by race/ethnicity demonstrated in this study, and the way in which these relationships varied by education level, provided potentially-valuable details about sub-populations of concussed patients who might be at increased risk for poor recovery. This knowledge could be used to help personalize treatment (e.g., with culturally-effective TBI education and mental health literacy) and symptom monitoring for these individuals. It was well established that education by providers had a profound impact on symptom improvement [63]. Although further research was needed to delineate the mechanisms of the significant relationships found in this study, patients might benefit from continued education during clinical visits to promote the importance of mental health in recovery, particularly among those who might have highly stigmatized views about seeking treatment for mental health issues. Provider training on culturally-sensitive clinical care [64], particularly on mental health, might also necessary to counter their unconscious bias, as well as to gain a better understanding of culturally-effective approaches to better address the needs of their patients, particularly those of racial/ethnic minorities.

### 4.1 Limitations

This study had limitations worth mentioning. First, the category of “non-Whites” included different races/ethnicities who might or might not share cultural similarities or physical traits that would affect sociopolitical hierarchy. As such, the effect sizes found in this study might not adequately represent all race/ethnic groups. Future studies on this topic might benefit from having a larger sample size in which race/ethnic groups could be evaluated at a more granular level, and could provide information on different social experiences that each race/ethnic groups might experience that could affect health outcomes such as mental health. Additionally, there might be differences in the validity of the instruments used in this study across the race/ethnic groups (i.e., measurement invariance). Thus, further studies on the validity of these instruments to measure depressive, PTSD and neurobehavioral symptoms among different race/ethnic groups were warranted. Another limitation was that education was categorized as a binary variable. Although this was based on a meaningful cutoff, it might not fully capture all important impacts of education on the relationship between race/ethnicity and mental health/neurobehavioral symptoms. Further, the type of education, knowledge or attitude (i.e., one that minimizes self-stigma and/or promotes necessary mental health treatment/assistance) might be more relevant to this association. Additionally, all variables used in this study were obtained via self-report, which was inherently impacted by certain biases (e.g., recall/reporting bias). However, such surveys afford quick assessments which might be more feasible than lengthier assessments, and outcome measures were based on well-validated instruments. Post-treatment data were also defined as the last follow-up available which could be prior to discharge data and, thus, may not reflect the full impact of treatment. Although we accounted for the length of treatment in all models, symptoms might be lower for “true” discharge date. Further, although it would be valuable to assess the effect of the number and type of specific previous and current treatment provided to each participant, this data was not available for analyses. This might have implications when studying racial/ethnic differences in symptom outcomes, specifically, whether different race/ethnic groups received differential treatments. Additionally, income was not available in the data used for this study. Although healthcare available at the TBI clinics were accessible for all service members, those with higher household income might have the financial capability to seek additional care external to the military health system. This study also did not statistically adjust for multiple comparisons as our sample size was limited. Future studies that investigate our relationships using a larger population were warranted.

## 5 Conclusions

The military healthcare system may need to increase depression-focused treatment options that are acceptable for racial/ethnic minority patients particularly with higher education while they are recovering from comorbid concussion.

## Funding

This study was supported by the 10.13039/100009898Defense Health Agency Traumatic Brain Injury Center of Excellence (Grant#: HT0014-21-C-0012).

## Disclaimers

The views expressed in this manuscript are those of the authors and do not necessarily represent the official policy or position of the Defense Health Agency, Department of Defense, or any other U.S. government agency. This work was prepared under Contract HT0014–21-C-0012 with DHA Contracting Office (CO-NCR) HT0014 and, therefore, is defined as U.S. Government work under Title 17 U.S.C.§101. Per Title 17 U.S.C.§105, copyright protection is not available for any work of the U.S. Government. For more information, please contact dha.TBICOEinfo@mail.mil.

## Acknowledgements and funding

The authors of this article would like to thank the service members who provided the data used in this study, as well as the clinical providers who served them. We would also like to recognize the contribution of the clinical staff at the Naval Hospital Camp Pendelton Intrepid Spirit Center 7 and the Naval Medical Center at San Diego so that studies such as this are possible.

## Declaration of Competing Interest

The authors declare no conflicts of interest.

## Appendix A Supplementary data

Supplementary material Image 1

## References

[1] Centers for Disease Control and Prevention (2016). 2016 Traumatic Brain Injury & Concussion, Report to Congress: Traumatic Brain Injury in the United States. https://www.cdc.gov/traumaticbraininjury/pubs/tbi_report_to_congress.html#:~:text=The%20annual%20unadjusted%20incidence%20rate,combined%20was%2075.5%20per%20100%2C000.

[2] Stein M.B., Jain S., Giacino J.T., Levin H., Dikmen S., Nelson L.D. (2019). Risk of posttraumatic stress disorder and major depression in civilian patients after mild traumatic brain injury: a TRACK-TBI Study. JAMA Psychiat.

[3] Department of Defense DoD TBI Worldwide Numbers. https://health.mil/Military-Health-Topics/Centers-of-Excellence/Traumatic-Brain-Injury-Center-of-Excellence/DOD-TBI-Worldwide-Numbers;.

[4] Liu Y., Collins C., Wang K., Xie X., Bie R. (2019). The prevalence and trend of depression among veterans in the United States. J Affect Disord.

[5] Gates M.A., Holowka D.W., Vasterling J.J., Keane T.M., Marx B.P., Rosen R.C. (2012). Posttraumatic stress disorder in veterans and military personnel: epidemiology, screening, and case recognition. Psychol Serv.

[6] Pew Research Center (2019). 2019 The changing profile of the US military: smaller in size, more diverse, more women in leadership. https://www.pewresearch.org/fact-tank/2019/09/10/the-changing-profile-of-the-u-s-military/.

[7] Arango-Lasprilla J.C., Ketchum J.M., Drew A., Hammond F., Powell J.M., Kreutzer J. (2012). Neurobehavioural symptoms 1 year after traumatic brain injury: a preliminary study of the relationshp between race/ethnicity and symptoms. Brain Inj.

[8] Arango-Lasprilla J.C., Kreutzer J.S. (2010). Racial and ethnic disparities in functional, psychosocial, and neurobehavioral outcomes after brain injury. J Head Trauma Rehabil.

[9] American Psychiatric Association (1994).

[10] Greenspan A.I., Stringer A.Y., Phillips V.L., Hammond F.M., Goldstein F.C. (2006). Symptoms of post-traumatic stress: intrusion and avoidance 6 and 12 months after TBI. Brain Inj.

[11] Sussman L.K., Robins L.N., Earls F. (1987). Treatment-seeking for depression by Black and White Americans. Soc Sci Med.

[12] Zhang A.Y., Snowden L.R., Sue S. (1998). Differences between Asian and White Americans’ help-seeking and utilization patterns in the Los Angeles area. J Community Psychol.

[13] McGuire T.G., Miranda J. (2008). New evidence regarding racial and ethnic disparities in mental health: policy implications. Health Affairs (Millwood).

[14] Office of the Surgeon General (US); Center for Mental Health Services (US): National Institute of Mental Health (US), Mental health care for African Americans (2001).

[15] Office of the Surgeon General (US); Center for Mental Health Services (US): National Institute of Mental Health (US), Mental health care for Asian Americans and Pacific Islanders (2001).

[16] Breslau J., Kendler K.S., Su M., Gaxiola-Aquilar S., Kessler R.C. (2005). Lifetime risk and persistence of psychiatric disorders across ethnic groups in the United States. Psychol Med.

[17] Williams D.R., Gonzalez H.M., Neighbors H., Nesse R., Abelson J.M., Sweetman J. (2007). Prevalence and distribution of major depressive disorder in African Americans, Carribean Blacks, and Non-Hispanic Whites: results from the National Survey of American Life. Arch Gen Psychiatry.

[18] Jorge R.E., Robinson R.G., Starkstein S.E., Arndt S.V. (1994). Influence of major depression on 1-year outcome in patients with traumatic brain injury. J Neurosurg.

[19] Dohrenwend B.P., Turner J.B., Turse N.A., Lewis-Fernandez R., Yager T.J. (2008). War-related posttraumatic stress disorder in Black, Hispanic, and majority White Vietnam veterans: the roles of exposure and vulnerability. J Trauma Stress.

[20] Koo K.H., Hebenstreit C.L., Madden E., Maguen S. (2016). PTSD detection and symptom presentation: racial/ethnic differences by gender among veterans with PTSD returning from Iraq and Afghanistan. J Affect Disord.

[21] Sripada R.K., Pfeiffer P.N., Rampton J., Ganoczy D., Rauch S.A.M., Polusny M.A. (2017). Predictors of PTSD symptom change among outpatients in the US Department of Veterans Affairs Health Care System. J Trauma Stress.

[22] Gary K.W., Arango-Lasprilla J.C., Stevens L.F. (2009). Do racial/ethnic differences exist in post-injury outcomes after TBI? A comprehensive review of the literature. Brain Inj.

[23] Shafi S., de la Plata C.M., Diaz-Arrastia R., Bransky A., Frankel H., Elliott A.C. (2007). Ethnic disparities exist in trauma care. J Trauma.

[24] Arango-Lasprilla J.C., Ketcham J.M., Lewis A.N., Krch D., Gary K.W., Dodd B.A. (2011). Racial and ethnic disparities in employment outcomes for persons with traumatic brain injury: a longitudinal investigation 1-5 years after injury. PM&R.

[25] Meagher A.D., Beadles C.A., Doorey J., Charles A.G. (2015). Racial and ethnic disparities in discharge to rehabilitation following traumatic brain injury. J Neurosurg.

[26] Schiraldi M., Patil C.G., Mukherjee D., Ugiliweneza B., Nuno M., Lad S.P. (2015). Effect of insurance and racial disparities on outcomes in traumatic brain injury. J Neurol Surg A Cent Eur Neurosurg.

[27] Asemota A.O., George B.P., Cumpsty-Fowler C.J., Haider A.H., Schneider E.B. (2013). Race and insurance disparities in discharge to rehabilitation for patients with traumatic brain injury. J Neurotrauma.

[28] Fuentes A., Schoen C., Kulzer R.R., Long C., Bushnik T., Rath J.F. (2019). Impact of racial-ethnic minority status and systemic vulnerabilities on time to acute TBI rehabilitation admission in an urban public hospital setting. Rehabil Psychol.

[29] Burnett D.M., Kolakowsky-Hayner S.A., Slater D., Stringer A., Bushnik T., Zafonte R. (2003). Ethnographic analysis of traumatic brain injury patients in the national Model Systems database. Arch Phys Med Rehabil.

[30] Lo L.L.H., Suen Y.N., Chan S.K.W., Sum M.Y., Charlton C., Hui C.L.M. (2021). Sociodemographic correlates of public stigma about mental illness: a population study on Hong Kong's Chinese population. BMC Psychiatry.

[31] Whaley A.L. (1997). Ethnic and racial differences in perceptions of dangerousness of persons with mental illness. Psychiatr Serv.

[32] Corrigan J.D., Bogner J. (2007). Initial reliability and validity of the Ohio State University TBI Identification Method. J Head Trauma Rehabil.

[33] American Psychological Association - APA Task Force on Race and Ethnicity Guidelines in Psychology (2019). 2019 APA Guidelines on Race and Ethnicity in Psychology. https://www.apa.org/about/policy/guidelines-race-ethnicity.pdf.

[34] Pressler S.J., Subramanian U., Perkins S.M., Gradus-Pizlo I., Kareken D., Kim J. (2011). Measuring depressive symptoms in heart failure: validity and reliability of the patient health questionnaire-8. Am J Crit Care.

[35] Zuromski K.L., Ustun B., Hwang I., Keane T.M., Marx B.P., Stein M.B. (2019). Developing an optimal short-form of the PTSD checklist for DSM-5 (PCL-5). Depress Anxiety.

[36] Blevins C.A., Weathers F.W., Davis M.T., Witte T.K., Domino J.L. (2015). The post-traumatic stress disorder checklist for DSM-5 (PCL-5): development and initial psychometric evaluation. J Trauma Stress.

[37] Cicerone K.D., Kalmar K. (1995). Persistent postconcussion syndrome: the structure of subjective complaints after mild traumatic brain injury. J Head Trauma Rehabil.

[38] King P.R., Donnelly K.T., Donnelly J.P., Dunnam M., Warner G., Kittleson C.J. (2012). Psychometric study of the Neurobehavioral Symptom Inventory. J Rehabil Res Dev.

[39] McGuire L.C., Strine T.W., Allen R.S., Anderson L.A., Mokdad A.H. (2009). The Patient Health Questionnaire 8: current depressive symptoms among U.S. older adults, 2006 Behavioral Risk Factor Surveillance System. Am J Geriatr Psychiatry.

[40] Weathers F.W., Litz B.T., Keane T.M., Palmieri P.A., Marx B.P., Schnurr P.P. (2013).

[41] Soble J.R., Silva M.A., Vanderploeg R.D., Curtiss G., Belanger H.G., Donnell A.J. (2014). Normative data for the Neurobehavioral Symptom Inventory (NSI) and post-concussion symptom profiles among TBI, PTSD, and nonclinical samples. Clin Neuropsychol.

[42] Belanger H.G., Lange R.T., Bailie J., Iverson G.L., Arrieux J.P., Ivins B.J. (2016). Interpreting change on the Neurobehavioral Symptom Inventory and the PTSD Checklist in military personnel. Clin Neuropsychol.

[43] Kroenke K. (2012). Enhancing the clinical utility of depression screening. CMAJ.

[44] Zou G. (2004). A modified Poisson regression approach to prospective studies with binary data. Am J Epidemiol.

[45] Cooper-Patrick L., Powe N.R., Jenckes M.W., Gonzales J.J., Levine D.M., Ford D.E. (1997). Identification of patient attitudes and preferences regarding treatment of depression. J Gen Intern Med.

[46] Wallen J. (1992). Providing culturally appropriate mental health services for minorities. J Ment Health Adm.

[47] Brown C., Conner K.O., Copeland V.C., Grote N., Beach S., Battista D. (2010). Depression stigma, race, and treatment seeking behavior and attitudes. J Community Pychol.

[48] Neighbors N.W. (1988). The help-seeking behavior of Black Americans. A summary of findings from the National Survey of Black Americans. J Natl Med Assoc.

[49] Sharp M-L., Fear N.T., Rona R.J., Wessely S., Greenberg N., Jones N. (2015). Stigma as a barrier to seeking health care among military personnel with mental health problems. Epidemiol Rev.

[50] Eylem O., de Wit L., van Straten A., Steubl L., Melissourgaki Z., Danisman G.T. (2000). Stigma for common mental disorders in racial minorities and majorities a systematic review and meta-analysis. BMC Public Health.

[51] Butler J.S. (1976). Inequality in the military: an examination of promotion time for Black and White enlisted men. Am Sociol Rev.

[52] Kales H.C., Blow F.C., Bingham C.R., Roberts J.S., Copeland L.A., Mellow A.M. (2000). Race, psychiatric diagnosis, and mental health care utilization in older patients. Am J Geriatr Psychiatry.

[53] van Ryn M., Fu S.S. (2003). Paved with good intentions: do public health and human service providers contribute to racial/ethnic disparities in health. Am J Public Health.

[54] Perez-Stable E.J., El-Toukhy S. (2018). Communicating with diverse patients: how patients and clinician factors affect disparities. Patient Educ Couns.

[55] van Ryn M., Burke J. (2000). The effect of patient race and socio-economic status on physicians’ perceptions of patients. Soc Sci Med.

[56] Schulman K.A., Berlin J.A., Harless W., Kerner J.F., Sistrunk S., Gersh B.J. (1999). The effect of race and sex on physicians' recommendations for cardiac catheterization. N Engl J Med.

[57] National Academies of Sciences, and Medicine; Health and Medicine Division; Board on Health Care Services; Committee to Evaluate the Department of Veterans Affairs Mental Health Services (2018). Evaluation of the Department of Veterans Affairs Mental Health Services.

[58] US Department of Defense (2020). https://www.defense.gov/News/News-Stories/Article/Article/2455474/air-force-releases-findings-of-racial-disparity-review/.

[59] Smith R.A., Applegate A. (2018). Mental health stigma and communication and their intersections with education. Commun Educ.

[60] Lopez V., Sanchez K., Killian M.O., Eghaneyan B.H. (2018). Depression screening and education: an examination of mental health literacy and stigma in a sample of Hispanic women. BMC Public Health.

[61] Guerra-Carrillo B., Katovich K., Bunge S.A. (2017). Does higher education hone cognitive functioning and learning efficacy? Findings from a large and diverse sample. PLoS One.

[62] Berkman N.D., Sheridan S.L., Donahue K.E., Halpern D.J., Crotty K. (2011). Low health literacy and health outcomes: an updated systematic review. Ann Intern Med.

[63] Paterick T.E., Patel N., Tajik A.J., Chandrasekaran K. (2017). Improving health outcomes through patient education and partnerships with patients. Proc (Baylor Univ Med Cent).

[64] Beach M.C., Price E.G., Gary T.L., Robinson K.A., Gozu A., Palacio A. (2005). Cultural competency: a systematic review of health care provider educational interventions. Med Care.

