# Supplementary material for: Disparities in mental health symptoms recovery across race/ethnicity and education level following mild traumatic brain injury
Source: Dialogues Health. 2022 Sep 24;1:100048. doi: 10.1016/j.dialog.2022.100048 (PMC10953859; doi:10.1016/j.dialog.2022.100048)
Supplement: Supplementary file 1 — Supplementary material [file mmc1.docx]

**Supplemental Table 1. Adjusted^a^ Overall Prevalence Ratio (PR [CI]) of Reaching a Clinically-meaningful PHQ-8 and PCL-5 Threshold Score by Race and Interaction By Education**

| **Outcomes** | **Race, n (%)** | | **Main Effects Adjusted PR (CI)** | **p-interaction with Education Level** |
| --- | --- | --- | --- | --- |
|  | **White** | **Non-White** |  |  |
| **PHQ-8** |  |  |  |  |
| **Clinically-elevated Pre-treatment (n=525)** |  |  | 0.94 (0.78, 1.14) | 0.307 |
| No | 248 (72.1) | 111 (61.3) |  |  |
| Yes | 96 (27.9) | 70 (38.7) |  |  |
| **Clinically-elevated Post-treatment (n=525)** |  |  | **^b^** | **0.002*** |
| No | 271 (78.8) | 119 (65.8) |  |  |
| Yes | 73 (21.2) | 62 (34.3) |  |  |
| **Clinically-relevant Increase (n=362)** |  |  | 1.18 (0.68, 2.03) | 0.169 |
| No | 207 (87.7) | 106 (84.1) |  |  |
| Yes | 29 (12.3) | 20 (15.9) |  |  |
| **Clinically-relevant Decrease (n=476)** |  |  | 0.88 (0.67, 1.15) | 0.278 |
| No | 207 (65.7) | 106 (65.8) |  |  |
| Yes | 108 (34.3) | 55 (34.2) |  |  |
| **PCL-5** |  |  |  |  |
| **Clinically-elevated Pre-treatment (n=546)** |  |  | 1.08 (0.89, 1.31) | 0.879 |
| No | 258 (71.5) | 96 (51.9) |  |  |
| Yes | 103 (28.5) | 89 (48.1) |  |  |
| **Clinically-elevated Post-treatment (n=546)** |  |  | ^b^ | **0.035*** |
| No | 263 (72.9) | 108 (58.4) |  |  |
| Yes | 98 (27.2) | 77 (41.6) |  |  |
| **Clinically-relevant** **Increase (n=322)** |  |  | 1.06 (0.76, 1.48) | 0.161 |
| No | 142 (66.7) | 67 (61.5) |  |  |
| Yes | 71 (33.3) | 42 (38.5) |  |  |
| **Clinically-relevant Decrease (n=433)** |  |  | 1.01 (0.83, 1.23) | 0.543 |
| No | 142 (49.0) | 67 (46.9) |  |  |
| Yes | 148 (51.0) | 76 (53.2) |  |  |

PHQ-8=Patient Health Questionnaire (8 items); PCL-5=Post-traumatic Stress Disorder Checklist, DSM-V

Cut-offs: Clinically-elevated PHQ-8 score=15, clinically-relevant increase/decrease in PHQ-8 score>=5; Clinically-elevated PCL-5 score=38, clinically-relevant increase/decrease in PCL-5 score>=7

^a^ Adjusted for study site, marital status, education (with vs. without college degree), primary language, branch of service, rank, number of days in the study, baseline outcome measure

^b^ Not available as the association varies by education level.

*Significant p-value at a level of 0.05.

**Supplemental Table 2. Adjusted^a^ Overall Prevalence Ratio (PR [CI]) of Reaching a Clinically-meaningful NSI (Total and By Subdomains) Threshold Score by Race and Interaction By Education**

| **NSI Outcomes** | **Race, n (%)** | | **Main Effects Adjusted PR (CI)** | **p-interaction with Education Level** |
| --- | --- | --- | --- | --- |
|  | **White** | **Non-White** |  |  |
| ***TOTAL*** |  |  |  |  |
| **Clinically-elevated Pre-treatment (n=545)** |  |  | 0.97 (0.94, 1.01) | 0.868 |
| No | 9 (2.5) | 7 (3.8) |  |  |
| Yes | 350 (97.5) | 179 (96.2) |  |  |
| **Clinically-elevated Post-treatment (n=545)** |  |  | 1.04 (0.97, 1.12) | 0.932 |
| No | 65 (18.1) | 20 (10.8) |  |  |
| Yes | 294 (81.9) | 166 (89.3) |  |  |
| **Clinically-relevant Increase (n=270)** |  |  | ^b^ | **0.040*** |
| No | 129 (75.9) | 70 (70.0) |  |  |
| Yes | 41 (24.1) | 30 (30.0) |  |  |
| **Clinically-relevant Decrease (n=474)** |  |  | 0.91 (0.77, 1.07) | 0.685 |
| No | 129 (40.6) | 70 (44.9) |  |  |
| Yes | 189 (59.4) | 86 (55.1) |  |  |
| ***CATEGORIES*** |  |  |  |  |
| **Pre-treatment** |  |  |  |  |
| **Clinically-elevated Cognitive Symptoms** |  |  | 0.99 (0.96, 1.02) | 0.965 |
| No | 8 (2.2) | 5 (2.7) |  |  |
| Yes | 351 (97.8) | 181 (97.3) |  |  |
| **Clinically-elevated Vestibular Symptoms** |  |  | Not calculable | Not calculable |
| No | 0 (0) | 0 (0) |  |  |
| Yes | 359 (100) | 186 (100) |  |  |
| **Clinically-elevated Somatosensory Symptoms** |  |  | 0.98 (0.93, 1.03) | 0.682 |
| No | 28 (7.8) | 12 (6.5) |  |  |
| Yes | 331 (92.2) | 174 (93.6) |  |  |
| **Clinically-elevated Affective Symptoms** |  |  | **0.94 (0.90, 0.99)*** | 0.398 |
| No | 18 (5.0) | 15 (8.1) |  |  |
| Yes | 341 (95.0) | 171 (91.9) |  |  |
| **Post-treatment** |  |  |  |  |
| **Clinically-elevated Cognitive Symptoms** |  |  | 1.02 (0.97, 1.07) | 0.451 |
| No | 39 (10.9) | 11 (5.9) |  |  |
| Yes | 320 (89.1) | 175 (94.1) |  |  |
| **Clinically-elevated Vestibular Symptoms** |  |  | Not calculable | Not calculable |
| No | 0 (0) | 1 (0.5) |  |  |
| Yes | 359 (100) | 185 (99.5) |  |  |
| **Clinically-elevated Somatosensory Symptoms** |  |  | 1.01 (0.93, 1.10) | 0.184 |
| No | 85 (23.7) | 29 (15.6) |  |  |
| Yes | 274 (76.3) | 157 (84.4) |  |  |
| **Clinically-elevated Affective Symptoms** |  |  | 1.03 (0.95, 1.11) | 0.414 |
| No | 88 (24.5) | 25 (13.4) |  |  |
| Yes | 271 (75.5) | 161 (86.6) |  |  |

NSI=Neurobehavioral Symptom Inventory

Cut-offs: Clinically-elevated NSI score (total and by categories) =75^th^ percentile of NSI (total and by categories), Clinically-relevant increase/decrease in NSI score=8

^a^ Adjusted for study site, marital status, education (with vs. without college degree), primary language, branch of service, rank, number of days in the study, baseline outcome measure

^b^ Not available as the association varies by education level.

*Significant p-value at a level of 0.05.
